# Transcriptomic Characterization of Candidate Genes for *Fusarium* Resistance in Maize (*Zea mays* L.)

**DOI:** 10.3390/pathogens14080779

**Published:** 2025-08-06

**Authors:** Aleksandra Sobiech, Agnieszka Tomkowiak, Tomasz Jamruszka, Tomasz Kosiada, Julia Spychała, Maciej Lenort, Jan Bocianowski

**Affiliations:** 1 Plant Breeding and Acclimatization Institute—National Research Institute in Radzików, 05-870 Błonie, Poland; j.spychala@ihar.edu.pl; 2 Department of Genetics and Plant Breeding, Faculty of Agronomy, Horticulture and Biotechnology, Poznań University of Life Sciences, Dojazd 11, 60-632 Poznań, Poland; agnieszka.tomkowiak@up.poznan.pl (A.T.); tomasz.jamruszka@up.poznan.pl (T.J.); maciej.lenort@up.poznan.pl (M.L.); 3 Department of Phytopathology, Seed Science and Technology, Faculty of Agronomy, Horticulture and Bioengineering, Poznań University of Life Sciences, Dąbrowskiego 159, 60-594 Poznań, Poland; tomasz.kosiada@up.poznan.pl; 4 Department of Mathematical and Statistical Methods, Poznań University of Life Sciences, Wojska Polskiego 28, 60-637 Poznań, Poland

**Keywords:** candidate genes, fusarium, plant resistance

## Abstract

Fusarium diseases are among the most dangerous fungal diseases of plants. To date, there are no plant protectants that completely prevent fusariosis. Current breeding trends are therefore focused on increasing genetic resistance. While global modern maize breeding relies on various molecular genetics techniques, they are useless without a precise characterization of genomic regions that determine plant physiological responses to fungi. The aim of this study was thus to characterize the expression of candidate genes that were previously reported by our team as harboring markers linked to fusarium resistance in maize. The plant material included one susceptible and four resistant varieties. Biotic stress was induced in adult plants by inoculation with fungal spores under controlled conditions. qRT-PCR was performed. The analysis focused on four genes that encode for GDSL esterase/lipase (LOC100273960), putrescine hydroxycinnamyltransferase (LOC103649226), peroxidase 72 (LOC100282124), and uncharacterized protein (LOC100501166). Their expression showed differences between analyzed time points and varieties, peaking at 6 hpi. The resistant varieties consistently showed higher levels of expression compared to the susceptible variety, indicating their stronger defense responses. Moreover, to better understand the function of these genes, their expression in various organs and tissues was also evaluated using publicly available transcriptomic data. Our results are consistent with literature reports that clearly indicate the involvement of these genes in the resistance response to fusarium. Thus, they further emphasize the high usefulness of the previously selected markers in breeding programs to select fusarium-resistant maize genotypes.

## 1. Introduction

Maize (*Zea mays* L.), along with wheat and rice, is the world’s leading staple cereal, with each grown on approximately 160 million hectares [1]. Since its domestication more than 9000 years ago in southern Mexico or Central America, maize has spread worldwide and has become the world’s leading staple grain in terms of annual production [1,2,3]. In 2022, the global maize harvest was about 1.15 billion tons at a yield of 5.82 t ha^−2^ [4].

In addition to providing nutrients for humans and animals, maize is a source for various other crucial products such as starch, oil, alcoholic beverages, food sweeteners, and fuel [5]. Unfortunately, the widespread use of this crop makes it vulnerable to pathogen destruction, resulting in huge losses. Fungal pathogens are among the main culprits.

Fungi of the genus *Fusarium* spp. are commonly found in maize crops and can cause a wide range of diseases, including root rot, ear rot, and stalk rot [5]. *Fusariosis* leads to reduced yields and grain quality [6]. The most common *Fusarium* species causing these diseases in temperate regions are *Fusarium graminearum*, *F. verticillioides*, and *F. subglutinans* [7]. They produce various toxins, including deoxynivalenol, zearalenone, and fumonisin [7,8]. These compounds can suppress the defense response of the infected plant by inhibiting cell division and protein synthesis. Climatic conditions, along with the genetic predisposition of maize varieties and production practices, are the three main factors influencing *Fusarium* infection and mycotoxin biosynthesis in maize. Nevertheless, climatic conditions appear to play a key role in the development of these pathogens. The geographic location of crops, and in particular the prevailing temperature and humidity, has a decisive influence on the intensity of *Fusarium* infection of plants [9]. Worth mentioning, breeding for resistance to cob fusariosis in maize is also hampered by the high genetic variability of the disease.

A plant’s susceptibility to infection is largely determined by its genetic makeup. A good example is *Bt* maize, genetically modified to express insecticidal proteins derived from *Bacillus thuringiensis*. This modification also provides enhanced resistance to *Fusarium* because the control of insect pests minimizes the wounds that serve as entry points for the fungal pathogen [10,11]. Genetic analysis of maize showed considerable heterogeneity in resistance to cob fusariosis, a finding corroborated by Zila et al. [12] and Chen et al. [13] in studies of large maize inbred line populations, including those from tropical regions. However, despite the identification of several inbred lines with stable resistance to the disease, which offer valuable starting material for maize breeding [14], completely resistant varieties have not yet been obtained.

Fusarium resistance is a quantitative trait controlled by multiple QTLs [15,16]. Consequently, the most effective current method of combating this pathogen is the cultivation of varieties with genetically determined resistance at multiple QTLs [17,18]. High-resolution genotyping facilitates GWAS-based polymorphism detection linked to target phenotypes, enabling precise QTL analysis and fine mapping [19,20,21]. This approach is increasingly applied also to the research on markers linked to plant pathogen resistance [22].

Diversity Arrays Technology sequencing (DArTseq) is a next-generation sequencing (NGS) technique that reduces genome complexity using specifically chosen restriction enzymes. This reduction allows for sequencing of low-copy genomic regions, which are often the most informative (diversityarray.com) [23]. Such an approach allows for the simultaneous study of hundreds of molecular markers across the genome. Therefore, it can be used for genome mapping in plant breeding programs, especially in the context of studying traits with complex inheritance, as well as for analyzing genetic diversity and expanding information on the population structure of crop plants [24].

Sobiech et al. [25,26] used DArTseq to obtain a total of 81,602 molecular markers (53,031 SilicoDArT and 28,571 SNPs). Association mapping selected 2962 markers (321 SilicoDArT and 2641 SNPs) significantly associated with plant resistance to fusarium. Of these 2962 markers, 7 markers (SilicoDArT and SNPs) were selected as significant at the 0.001 level. They were used for physical mapping, indicating that four of them are intragenic. Marker 553 is anchored to the *GDSL esterase/lipase gene* (LOC100273960); marker 15,097 to the *putrescine hydroxycinnamyltransferase gene* (LOC103649226), marker 58,771 to the *peroxidase 72 gene* (LOC100282124), while marker 27,775 is anchored to the gene encoding for an *uncharacterized protein* (LOC100501166) [25,26].

GDSL esterase/lipase (GELP) proteins belong to the SGNH superfamily of hydrolases and contain a conserved GDSL motif at the N-terminus. More than 1100 members of the GDSL esterase/lipase family have been found in 12 fully sequenced plant genomes, and they have become very attractive topics due to their recently discovered properties and functions. Several plant GELPs have been isolated, cloned, and characterized. Members of the GELP family are primarily involved in the regulation of plant development, morphogenesis, secondary metabolite synthesis, and defense response [27,28,29,30,31,32,33,34,35,36,37,38,39,40,41,42,43,44,45,46,47].

In the present study, we focused on expression profiling of genes previously described by our team as harboring markers linked to fusarium resistance, namely the GDSL esterase/lipase gene (LOC100273960), putrescine hydroxycinnamyltransferase gene (LOC103649226), peroxidase 72 gene (LOC100282124), and a gene encoding for an uncharacterized protein (LOC100501166), using real-time PCR. One susceptible (FR) and four resistant (KF12, KF15, SF11, and SF12) maize varieties were analyzed. To confirm the presence of markers determining fusarium resistance in the tested genotypes, genomic DNA was isolated, and a PCR reaction was performed. Additionally, expression levels of these genes were also characterized with a use of publicly available transcriptomic data.

## 2. Materials and Methods

### 2.1. Material and Fusarium Inoculation Under Controlled Conditions

The plant material used in the research consisted of five maize genotypes. The susceptibility control (resistance to fusarium at level 4 on the COBORU scale, determined through phenotypic evaluations, where 0 indicates the highest susceptibility and 9 indicates the highest resistance) was the FR cultivar from the resources of the Department of Plant Genetics and Breeding at Poznań University of Life Sciences. The other four cultivars exhibited a fusarium resistance level of 9 on the COBORU scale. KF12 and KF15 varieties were obtained from Małopolska Plant Breeding Ltd. Co. in Kobierzyce, Poland (50°58′17″ N 16°55′50″ E), while varieties SF11 and SF12 from Plant Breeding Smolice Ltd. IHAR Group in Smolice, Poland (51°42′12″ N 17°10′10″ E).

*Fusarium* inoculation was conducted in a vegetation chamber under controlled conditions. The temperature was set at 22 °C during the day and 18 °C at night with a 16-h photoperiod. Inoculation was carried out in October 2023, right after *Fusarium* was harvested from the research plots. The relative humidity was between 60 and 70%. In addition, the emission spectrum of the light source was set with a photon flux of 572 μE. Four maize kernels of each genotype were sown into 16 cm diameter pots with soil from a cultivated field, in four replicates. The soil was kept at a moisture content of about 70% for the growth period. At the stage of 4–5 leaves, maize plants were artificially infected with a suspension of spores and mycelium of several fungal isolates of the genus *Fusarium* (*Fusarium graminearum* isolates Fg/D, *Fusarium boothii* isolate F0410/7, 20K, *Fusarium pseudograminearum* isolates: F2811, 1428/12b, *Fusarium subglutinans* ZK4). Fungal material collected from the kernels and gene bank (described in Section 3.1) was used to prepare the inoculate. Inoculation was performed by spraying a suspension of conidia at a concentration of about 5 × 105 spores mL^−1^. The inoculum suspension was an equimolar mixture of four *Fusarium* strains suspended in water with 1% *v*/*v* Tween 20 reagent and prepared immediately before inoculation. Two leaves from each combination were taken for further study. Leaf tissue sections were sampled at five time points: 0 (before inoculation), 6, 12, 24, and 72 hpi, in three biological replicates. Plants were immediately frozen and stored at −20 °C.

### 2.2. Material and Fusarium Genotype Analysis

#### Evaluation of the Species Affiliation of the Pathogenic Fungus

During the vegetative season, plant fragments were taken from maize plants with symptoms of fusariosis in order to isolate the pathogens causing the disease symptoms. The plant fragments were placed in 2% sodium hypochlorite for 1 min to disinfect the surface tissues. The plant fragments were then lined on PDA medium (potato-dextrose agar medium). After about 7 days, the grown cultures were transplanted to the new medium. Initial selection of the obtained cultures for belonging to the genus *Fusarium* was followed by culture on SNA medium (Saltwater Nutrient Agar) and then assessment of species affiliation by microscopy using commonly used keys [48,49,50]. DNA isolation material was prepared from the mononuclear cultures. After the DNA was isolated using the method of Doyle and Doyle [51], PCR was performed in order to confirm or verify the species affiliation of the obtained isolates of fungi of the genus *Fusarium*. The nuclear ribosomal internal transcribed spacer (ITS) regions were amplified. The analysis involved rDNA fragments capped by DNA fragments complementary to primers ITS1 (5′ TCCGTAGGTGAACCTGCGG 3′) and ITS4 (5′ TCCTCCGCTTATTGATATGC3′) [52], as well as fragments of the tef1 gene (encoding an elongating transcription factor) [53] and β-tub (encoding β-tubulin). The reaction was carried out with the following parameters: initial denaturation 95 °C—5 min, denaturation 94 °C—30 s, annealing 55 °C—70 s, elongation 72 °C—35 s, repeated 35 times, final elongation 72 °C—10 min. After the reaction, the DNA product was purified using a clean-up concentrator kit (A&A Biotechnology, Gdańsk, Poland) and sent to Macrogen Europe (Amsterdam, The Netherlands) for sequencing. The BLASTn program (http://blast.ncbi.nlm.nih.gov/) [54] and FUSARIUMID v. 1.0, a publicly available DNA database of the translation elongation factor 1-alpha (TEF) partial elongation factor, were used for sequence identification.

### 2.3. Selection of Candidate Genes and Identification of Associated Molecular Markers

Candidate genes were selected based on results previously published by Sobiech et al. [25,26], where Diversity Arrays Technology sequencing (DArTseq), association mapping, and physical mapping were used to identify markers and link candidate genes associated with fusarium resistance. In this publication, we examined the expression of these genes in resistant and susceptible genotypes to confirm their impact on fusarium resistance. We also tested whether specific molecular markers located in these candidate genes differentiate between resistant and susceptible genotypes and could be used for selection in breeding programs.

### 2.4. DNA Isolation

To confirm the presence of markers determining *Fusarium* resistance in the tested genotypes, their genomic DNA was isolated, and a PCR reaction was performed. DNA was isolated from leaves of 10-day-old seedlings using the Maxwell^®^ RSC Tissue DNA Kit (Promega, Madison, WI, USA), according to the manufacturer’s recommended procedure. DNA concentration and quality were determined using a DeNovix spectrophotometer (DeNovix Inc., Wilmington, DE, USA). Samples were diluted with buffer (Promega, Madison, WI, USA) to obtain a uniform concentration of 30 ng μL^−1^.

### 2.5. PCR Conditions

PCR primers were designed based on the marker sequences analyzed [25,26]. PCR was performed in a T1000 thermocycler (Bio-Rad, Hercules, CA, USA). The 20 μL reaction mixture contained 5xGreen GoTaq Flexi Reaction Buffer—4 μL; forward and reverse primers (10 μM)—0.5 μL each 25 mM MgCl_2_, 1.6 μL; 0.32 μL of 10 mM Ultrapure dNTPs Mix; 0.17 μL of GoTaq G2 Flexi DNA Polymerase (Promega, Madison, WA, USA); 10.91 μL of nuclease-free water; and 50 ng DNA—2 μL. PCR conditions were as follows: initial denaturation for 2 min at 94 °C, followed by 40 cycles; denaturation for 1 min at 95 °C; annealing at 50 s (Table 1); elongation for 1 min at 72 °C; final denaturation for 5 min at 72 °C; and holding for ∞ at 4 °C, for a total of 40 cycles. The primer sequences and annealing temperatures are shown in Table 1.

### 2.6. Electrophoresis

Electrophoresis of PCR products was carried out in a 2% agarose gel for 1.5 h at 110 V. First, 5 μL of Midori Green Advance DNA dye solution (NIPPON, Düren, Germany) was added per 100 mL of gel to visualize the DNA. PCR product size was assessed using a 50 bp or 100 bp DNA Step Ladder (Promega, Madison, WI, USA). Visualization of separated DNA fragments in the gel by UV light was performed using the Gel Doc XR+ gel documentation system (Bio-Rad, Hercules, CA, USA).

### 2.7. Quantitative (Real-Time) PCR

#### 2.7.1. RNA Isolation

Isolation of total RNA from leaf tissue samples collected in 3 biological repeats at 5 different time points (0 and 6, 12, 24, 48 h post-inoculation) was carried out using the Maxwell RSC Plant RNA Kit (Promega, Madison, WI, USA). The concentration and purity of isolated total RNA were assessed using a NanoDrop spectrophotometer at an A260/A280 ratio. Synthesis of the first strand of cDNA was performed using the iScript™ Reverse Transcription Supermix kit for RT-PCR (Bio-Rad, Hercules, CA, USA), according to the manufacturer’s protocol.

#### 2.7.2. Primer Design and Conditions

Sequences of the candidate genes were found in the BLAST database and downloaded in FASTA format. The sequences were used to design primers for qRT-PCRs using Primer3Plus (Table 2).

For each primer pair, PCR was performed with a temperature gradient to optimize the thermal profile used. The gradient was set in the temperature range from 52 °C to 64 °C. The optimal amplification temperature was set at 60 °C after obtaining a specific product in a 2% agarose gel.

To perform amplification standard curves, individually obtained amplicons were purified using the QIAquick PCR Purification Kit (Qiagen Inc., Hilden, Germany) according to the company’s protocol. In the next step, a series of dilutions was performed. Dilutions for each gene tested were applied to a plate, and RT-PCR reactions were carried out. Expression analysis for this experiment was performed using Bio-Rad CFX Maestro software v. 2.3 and the GeneStudy tool (Bio-Rad, Hercules, CA, USA), obtaining normalized gene expression values.

qRT-PCR analyses were performed using iTaq Universal SYBR Green Supermix (Bio-Rad, Hercules, CA, USA) and CFX96 Touch Real-Time PCR Detection System (Bio-Rad, Hercules, CA, USA). Each of the qRT-PCR experiments performed consisted of three biological replicates and three technical replicates, the results of which were averaged. Simultaneously, for each tested gene, negative control without cDNA template—NTC (No-Template Control) was performed in three technical replicates. The composition of the qRT-PCR mixture was as follows: iTaq supermix—5 μL; forward and reverse primers (10 μM)—0.5 μL each; 3 μL of nuclease-free water; and cDNA template—1 μL. The following temperature profile was used in qRT-PCRs: initial denaturation for 3 min at 95 °C, followed by 40 cycles; denaturation for 10 s at 95 °C; and finally, annealing for 30 s at 60 °C. During the melting stage (melting curve), the temperature ranged from 65 °C to 90 °C; every 5 s, the temperature was increased by 0.5 °C. Expression analysis for this experiment was performed using Bio-Rad CFX Maestro software v. 2.3 and the GeneStudy tool (Bio-Rad, Hercules, CA, USA), obtaining normalized gene expression values. The expression results of the studied genes were related to the expression values of reference genes, which have a stable expression profile for a given experiment. This allowed normalized expression values of the studied genes to be obtained. The primer sequences and annealing temperature are shown in Table 2.

#### 2.7.3. Selection of Reference Genes for Quantitative PCR. Development of Standard Curves Genes

Based on the literature data, four reference genes for stable expression were selected: Actin2, Elongation factor 1/α-EC1α, β-tubulin, and cyclophilin [55,56] (Table 2). After analyzing the standard curves for these reference genes, two genes, β-tubulin (β-TUB) and cyclophilin (CYT), were selected, and qRT-PCR analyses were performed for them on the test cDNA template. The selection and testing of reference genes were developed according to the protocol described [57]. The analysis of reference genes for the test samples was necessary for statistical calculations, as the expression of the reference gene was compared with the expression of the gene analyzed for each sample using the Gene Study tool (CFXMaestro from Bio-Rad Laboratories, Inc., Hercules, CA, USA).

Elongation factor 1 alpha (EF1α), tubulin beta (β-TUB), cyclophilin (CYP), and eukaryotic initiation factor 4A (EIF4A) were chosen as the most suitable reference genes for normalizing gene expression using qRT-PCR in maize. Further validation using each of the most stable reference genes, EF1a and β-TUB, and their combination (EF1a + β-TUB) confirmed that EF1a and β-TUB were suitable reference genes for normalizing qRT-PCR data [56]. In contrast, during our analyses, we found that EF1 α was a much less effective reference gene than CYP.

### 2.8. Transcriptomics Analysis

Accession numbers used in transcriptomic data analysis were obtained from the National Center for Biotechnology Information (NCBI) website https://www.ncbi.nlm.nih.gov/ (accessed on 1 August 2024) [58]. The expression level of identified genes, measured as Fragments Per Kilobase of transcript per Million mapped reads (FPKM), in different Z. mays organs and tissues was determined based on transcriptomic data from Walley et al. [59]. The expression level of analyzed genes in various organs and tissues of *Z. mays* was assessed with a use of transcriptomic data from Walley et al. [59].

### 2.9. Statistical Analysis

The Kolmogorov–Smirnov test was used to test the null hypothesis that the empirical distribution of normalized expression data for a specific gene and line complied with the normal distribution. This test was applied to all gene × line combinations. To assess the homogeneity of variances, the Brown–Forsythe variant of Levene’s test was calculated (passed for all tested time point × gene × line combinations). For comparisons of time measurements, a repeated measures ANOVA was performed, and then *t*-tests were performed between the lines per time point to analyze the time and line interactions at the significance level of *p* < 0.05. The *p*-values were adjusted using Benjamini–Hochberg correction for multiple testing. Analyses were performed to assess the variability of the individual expression profiles of the analyzed genes of candidate genes F1, F2, F3, F4 (Table 3 and Table 4), as well as two reference genes: CYT and β-TUB. The relationship between gene expressions was assessed using Pearson’s linear correlation coefficient. For comparison of means, a two-sample Student’s *t*-test was performed. The expression of the analyzed candidate genes is shown as heatmaps. For visualization of gene expression results, cluster analysis (UPGMA method) with Euclidean distances was performed. The data were also subjected to multivariate statistical analyses, including canonical variable analysis [60] and Mahalanobis distances [61,62,63]. All these analyses were conducted using the Genstat v.23 statistical software package (VSN International 2023) [64].

## 3. Results

### 3.1. Colonization of Different Maize Varieties by Five Fusarium Species

**Figure 1:**
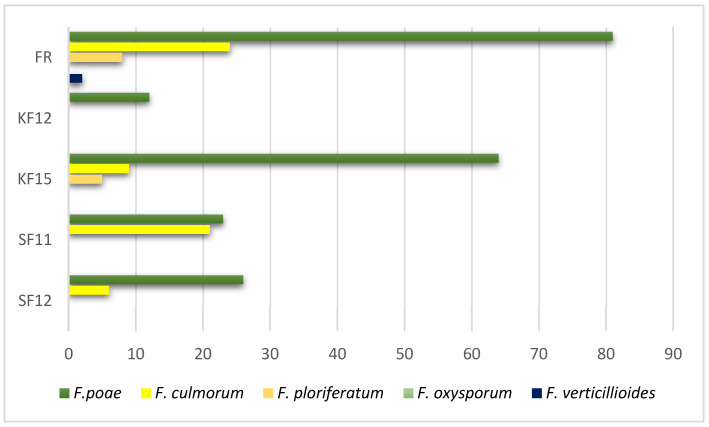
Inoculation of kernels with different genotypes of the genus fusarium of selected corn lines (FR—susceptible, and the other four—resistant). The *y*-axis shows the % of colonized kernels.

Assessment of kernels taken from the growing season showed varying levels of their colonization by fungi of the genus *Fusarium*. Among all samples, the most abundant fungi were *F. poae* (Figure 1).

### 3.2. Expression of Candidate Genes Linked to Fusiarium Resistance

Repeated-measure analysis of variance indicated that the main effects of line were significant for expression of all six genes, except F4 (Table 3). The main effects of the term were significant for expression of genes F2, F3, CYT, and TUB. Whereas, line x term interaction was significant for expression of only F3 (Table 3).

Changes in expression levels of four genes post-inoculation (in relation to samples before inoculation) are shown in Table 4. Since each gene had a specific expression level, analysis of variance was performed for each gene separately (Table 3). The varieties showed unique patterns of gene expression in response to *Fusarium* infection. Each gene responded differently to pathogen infection and differed in expression levels across varieties and time points.

**Figure 2:**
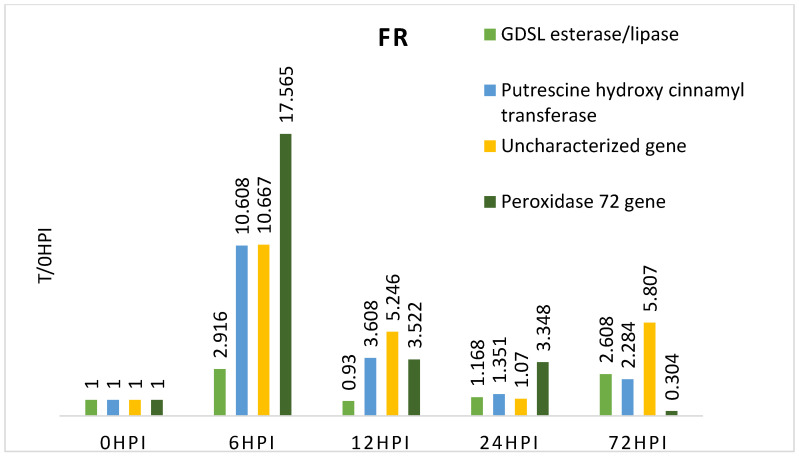
Expression level of the genes studied in relation to time 0 in the resistant maize genotype (relative to expression before inoculation—0 h).

The FR (Farm Modena) variety showed particularly high expression levels for all genes 6 h after inoculation. This may indicate the activation of immune responses early during the infection. In the following hours (12 h and 24 h) after inoculation, a decrease in the expression level of all genes tested in this variety was observed. Noteworthy is the fact that the gene F4, 72 h after inoculation, was characterized by lower expression than before inoculation. This indicates that the resistance of this gene was broken after this time, confirming that this cultivar is more susceptible to *Fusarium* spp. infection than the other genotypes and may be a negative control (Table 4, Figure 2).

**Figure 3:**
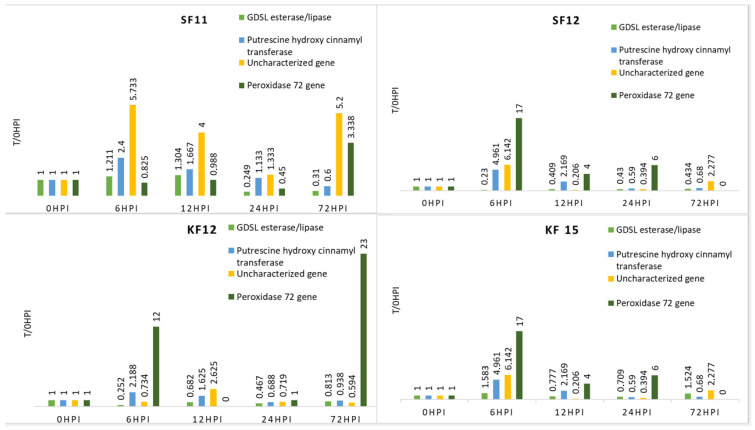
Expression level of the genes studied in relation to time 0 in 4 resistant maize genotypes (relative to expression before inoculation—0 h).

For the KF12 genotype, the F1, F2, and F3 genes showed varying levels of expression at different time points, except 72 h after inoculation. Finally, for these genes, the value of the expression level was lower than before inoculation. It should be noted that among all the analyzed genes, only F4 had a higher expression level 72 h after inoculation than before (Table 4, Figure 3).

In the case of the KF15 variety, it is worth noting that the F1 and F3 genes showed higher expression levels 72 h after inoculation than before it. At earlier time points, their expression levels varied. For the F2 and F4 genes, the expression level at 72 h after inoculation was lower than before (for the F4 gene, it was 0) (Table 4, Figure 3).

In the case of the SF11 cultivar, the highest expression was observed for the F3 gene, where at each time point its expression was higher than before inoculation. In contrast, the F2 gene reached its highest level of expression 6 h after inoculation, while for the F1 gene, the peak occurred after 12 h (Table 4, Figure 3).

The SF12 genotype was characterized by a significant increase in expression for the F4 gene 72 h after inoculation. The expression of the F1 gene was lower after inoculation at all tested times than before. For F2 and F3 genes, the highest expression occurred at 6 h after inoculation, then dropped below baseline (Table 4, Figure 3).

Interestingly, for all genes except F1, genotypes KF12 and KF15 (sourced from the Malopolska HR) had the most dissimilar expression pattern among themselves, but were the most similar among themselves. On the other hand, genotypes SF12 and SF11 (from HR Smolice) had the most similar expression pattern among themselves for the F2 gene (putrescine hydroxycinnamonotransferase) and the F1 gene (GDSL esterase/lipase At4g01130 uncharacterized precursor protein). This observation may indicate that genotypes from the same cultures have similar expression patterns (Figure 3).

**Figure 4:**
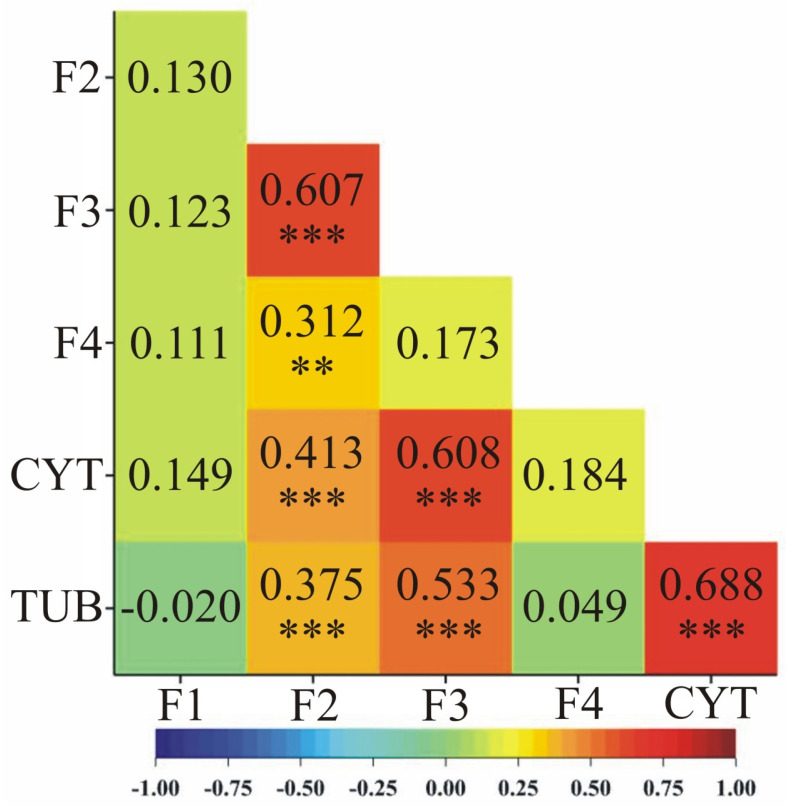
Heatmaps of Pearson’s linear pairwise correlation coefficients between the observed genes based on the values of their individual expression profiles. Significance of pairwise correlations is marked: ** *p* < 0.01, *** *p* < 0.001. Heatmap color scheme (labeled at the bottom) indicates more positive (red hue) or negative (blue hue) correlations.

A comparison of expression profiles between genes was also attempted by correlation analysis. Expression of candidate gene F2 was positively correlated with expression of candidate genes F3 (0.607) and F4 (0.312) (Figure 4).

**Figure 5:**
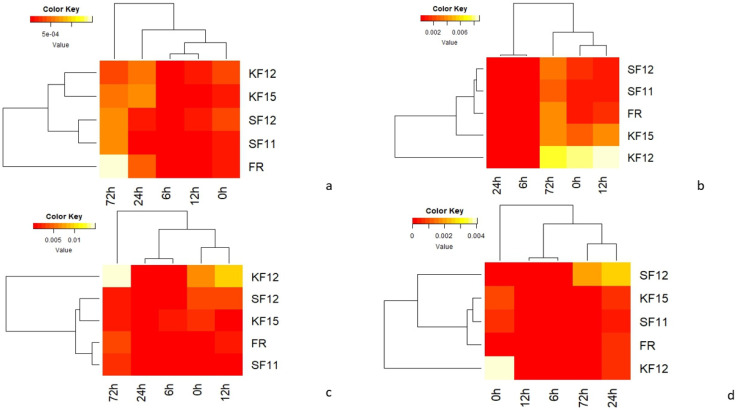
Heat maps showing expression profiles obtained using quantitative real-time PCR analysis; (**a**)—F1 gene (GDSL esterase/lipase gene At4g01130 precursor of uncharacterized precursor protein), (**b**)—F2 gene (putrescine hydroxycinnamotransferase, LOC103649226), (**c**)—F3 gene (uncharacterized protein), (**d**)—F4 gene (peroxidase 72 precursor gene and protein containing pentatriopeptide repeats At5g57250, mitochondrial).

The average values from qRT-PCR analysis of candidate genes in all maize genotypes tested at different time points were summarized as heat maps (Figure 5). For each cultivar tested, the strongest expression was observed for the *peroxidase 72 gene* (F4) and the uncharacterized gene (F3). In all resistant genotypes (KF12, K15, SF11, SF12), F4 gene activity remained high immediately after inoculation. In the SF12 genotype classified as resistant, the F4 gene showed low expression at 72 hpi, which may indicate that the resistance is not determined by this gene. In the FR variety, which is a susceptible variety, the activity of this gene significantly decreased at 72 hpi, which may indicate that the plant’s resistance has been broken (Figure 5).

**Figure 6:**
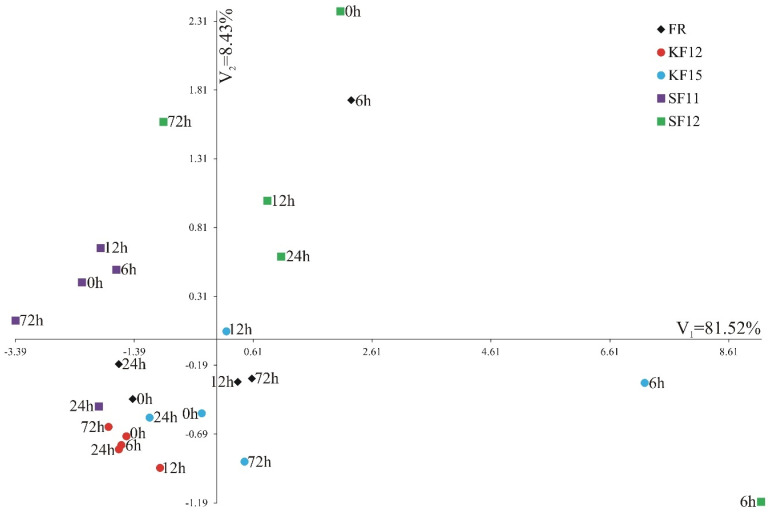
Distribution of the combinations of lines and terms of the system of the first two canonical variates: V_1_—first canonical variable, and V_2_—second canonical variable.

A comparison of the gene expression of combinations of lines and terms using multivariate methods is presented in Figure 6. Canonical variate analysis was performed to check whether the analyzed combinations of lines and terms were grouped depending on the expression level of individual genes. The first canonical variate explained 81.52% of the total line-by-term combinations variability, whereas the second canonical variate determined the line-by-term combinations variability in 8.43% (Figure 6). The greatest multidimensional similarity in terms of expression of four genes, by the Mahalanobis distance, was observed for KF12 at 6 h and KF12 at 24 hpi (0.15). The most diverse line-by-term combinations were SF11 at 72 h and SF12 at 6 hpi (12.703).

### 3.3. Identification of SilicoDArT Markers Linked to Candidate Genes Determining Fusarium Resistance in Maize

**Figure 7:**
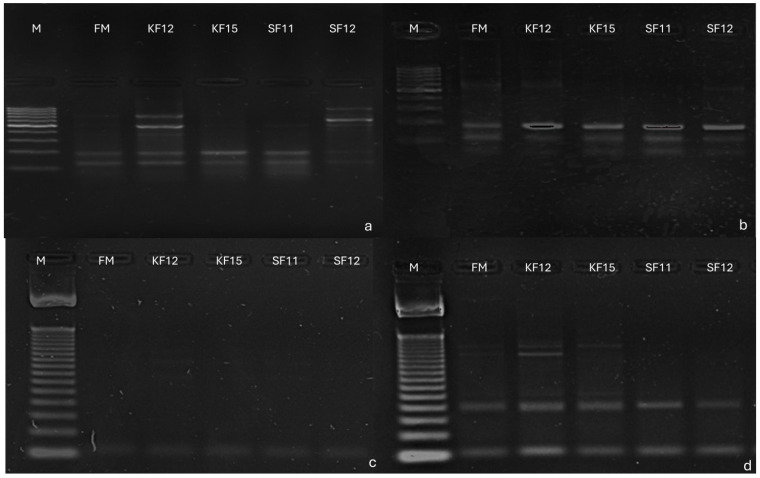
Electrophoregram illustrating: (**a**) amplification products for marker 553, (**b**) amplification products for marker 15,097, (**c**) amplification products for marker 58,771, (**d**) amplification products for marker 27,775. Ladder size 100 bp. Order of paths on the gel: 1 M—size marker, 2—FM genotype, 3—KF12 genotype, 4—KF15 genotype, 5—SF11 genotype, 6—SF12 genotype.

Using polymerase chain reaction (PCR), selected markers were tested on five genotypes. All markers showed the presence of their associated genes. In marker 553, a product occurred at 116 bp, indicating the presence of the gene GDSL esterase/lipase At4g01130 precursor, an uncharacterized precursor of the protein in all genotypes, despite the presence of non-specific products. It occurred in every genotype tested, but non-specific products occurred. At marker 15,097, product occurred at 107 bp in all genotypes, indicating the presence of in putrescine hydroxy cinnamyl transferase gene; however, non-specific products occurred. In marker 58,771, a product of 103 bp occurred in all genotypes, indicating the presence of peroxidase precursor gene 72 and pentatricopeptide repeat-containing protein At5g57250, mitochondrial, while in marker 27,775, a product of 167 bp confirms the presence of protein loc 100,501,166 despite the presence of non-specific products. It occurred in every genotype tested, but non-specific products occurred (Figure 7).

### 3.4. Transcriptomic Data Analysis

**Figure 8:**
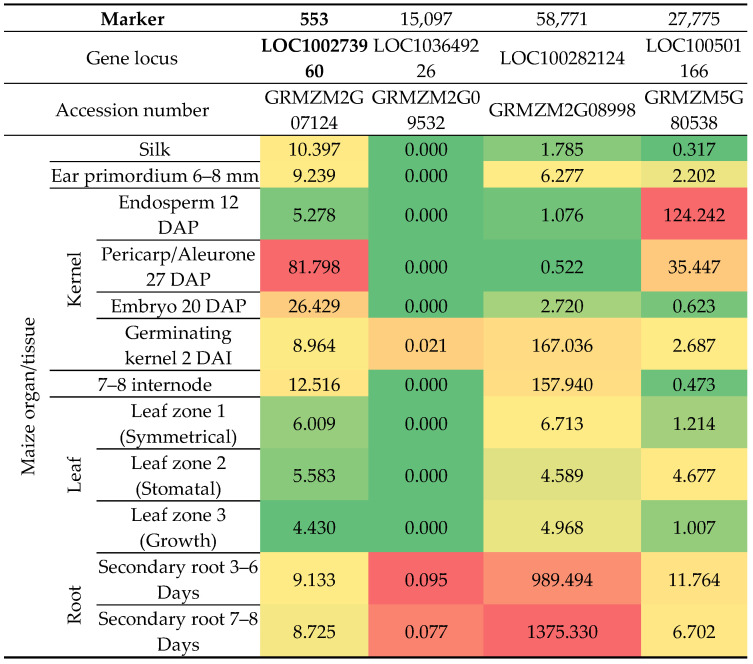
A heatmap showing changes in the expression levels of four genes that harbor selected markers in chosen *Z. mays* organs and tissues. Locus and accession numbers that enabled analysis of transcriptomic data are presented. The heatmap depicts Fragments Per Kilobase per Million mapped fragments (FPKM) values in transcriptomic data acquired from Walley et al. (2016) [59]. For each gene (columns) separately, the intensity of red indicates higher gene expression, the intensity of green indicates lower gene expression, while moderate gene expression values are marked in yellow-orange. In this study, changes in the expression levels of four genes carrying selected markers were traced. Their expression was additionally assessed in susceptible and resistant *Z. mays* genotypes infected by *F. verticillioides*. The loci and accession numbers that enabled the analysis of transcriptomic data are presented. Transcriptomic data obtained from Lanubile et al. [65] served as confirmation that the markers we selected differentiated between fusarium-resistant and susceptible genotypes (Figure 9). The analysis confirmed that a greater change in expression after inoculation with *F. verticillioides* occurred in fusarium-resistant genotypes in all selected markers except marker 553.

Two of these genes harboring 553 and 27,775 markers were expressed most strongly in kernel compartments, pericarp/aleurone and endosperm, respectively. The highest transcript abundance of genes with 15,097 and 58,771 markers was found in the root, while the expression level of genes with 58,771 markers was the highest in the leaf and ear primordium (Figure 8).

**Figure 9:**
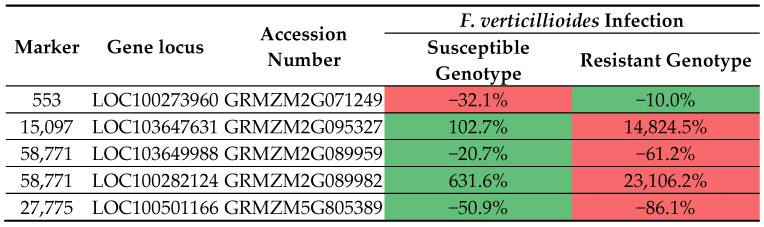
The heatmap depicts % changes of expression caused by *F. verticillioides* in relation to the non-infected control. For each gene, red indicates the genotype with more prominent change and green indicates the genotype with less prominent change in expression after *F. verticillioides* infection. To enable visualization of expression changes for the LOC103647631 gene, the control expression value in the resistant genotype was set from 0 to 0.001 FPKM.

## 4. Discussion

In recent years, there has been a strong emphasis on resistance breeding. Sobiech et al. [25] attempted to identify molecular markers associated with genes showing *Fusarium* resistance, while Tomkowiak et al. [66] attempted to identify molecular markers associated with genes showing smut (*Ustilago maydis* (D.C.) Corda) resistance.

Fungi of the genus *Fusarium* are among the most important pathogens of crop plants [67]. The importance of the genus is determined at least by the fact that they are pohyparasitic pathogens that can develop saprotrophically and produce different types of spores, including spores of a spore-survival nature—chlamydospores. Maize is infested by various species, including *F. graminearum*, *F. fujikuroi*, *F. subglutinans*, and *F. culmorum*. Symptoms can be observed on various parts of the plant, on the roots, stems, leaves, cobs, and grains. The problem in agriculture is not only the occurrence of the disease, resulting in reduced yields, but also the occurrence of mycotoxins (secondary metabolites of *Fusarium* fungi), which are harmful to humans and animals [68].

In a previous study, DArTseq technology using an NGS platform, combined with association and physical mapping, was used to identify molecular markers and associated candidate genes that determine resistance to fusarium in maize [25,26]. Four significant molecular markers of SNPs and SilicoDArT, namely 553, 15,097, 58,771, and 27,775, were initially selected.

These markers are located on chromosomes 9, 2, and 37, 3 within four genes that encode for GDSL esterase/lipase (LOC100273960), putrescine hydroxycinnamyltransferase (LOC103649226), peroxidase 72 (LOC100282124), and uncharacterized protein (LOC100501166), respectively. This publication attempts to characterize the above-mentioned candidate genes and evaluate their role in shaping fusarium resistance in maize. In addition, their expression was analyzed in reference materials of varieties with high resistance and susceptible varieties.

GDSL esterase/lipase (GELP) proteins are a very large subfamily of lipolytic enzymes, and have been identified in microorganisms and many plants, but only a few have been characterized for their role in growth, development, and stress response. Characterization and traits of GEL Pz genes have been analyzed at the genomic level in *Arabidopsis*, *Sorghum bicolour*, *Populus tricholarpa*, *Oryza sativa*, maize, and other plant species [69,70,71]. They play important roles in abiotic stress response, morphogenesis, and defense against pathogens [72]. Su et al. [73] found 194 GELP-encoding genes in the soybean genome and conducted a comprehensive analysis, including phylogenetic analysis, evolutionary traits, and expression profiles, identifying a candidate GELP gene, GmGELP28, involved in drought- and salt-tolerance.

To better understand the precise role of GELPs in plant development and stress response, and to facilitate the breeding of resistant crops to effectively secure the food supply, combined genomics with genetic, biochemical, and molecular approaches is needed [74].

Cotton GELPs showed diverse expression patterns in tissue development, ovule and fiber growth, and in response to biotic and abiotic stresses, linking existing cis-elements in promoter regions. Analysis of existing cis-elements in promoter regions suggests diverse GELP functions and hormone-dependent modes of action [75]. An et al. [76] identified and functionally characterized the GMS gene ZmMs30, which encodes a novel type of GDSL lipase and plays crucial roles in the aliphatic metabolism during maize anther and pollen development.

In our study, we found that the expression of the analyzed GDSL esterase/lipase gene increased after inoculation in almost all the cultivars tested, most notably in the cultivar with the highest resistance to *Fusarium*, so this may be a result of the plant’s response to stress associated with pathogen infection (Table 4). The results obtained allow us to conclude that this gene was linked to the mechanism of the resistance response to *Fusarium* infection in maize.

Polyamines aliphatic (PA)—organic compounds of low molar mass, having at least two amine groups in their structure—are found in the cells of animals (including humans), plants, and bacteria. They are classified as biogenic amines, as in the case of putrescine. Previous studies of Walters et al. [77] have shown that polyamines can play a key role in plant responses to pathogens. In plant cells, polyamines can occur in free or conjugated form with phenolic acids, mainly ferulic acid, p-coumaric acid, and caffeic acid (hydroxycinnamic acid amides), or bound to macromolecules, including proteins and cell wall components [77] Additionally, a few studies indicate the occurrence of relationships between polyamines and plant defense hormones during plant biotic stress, and that polyamines may interfere with ethylene, salicylic acid, and abscisic acid metabolisms and vice versa [78]. There are also a few investigations that addressed antifungal activities of free polyamines and hydoxycinnamic acid amines [77].

Wojtasik et al. [79] report increased expression levels of various genes involved in polyamine biosynthesis after flax infection by *F. graminearum*, leading to a significant increase in polyamine levels in plant tissues. Despite studies showing altered polyamine levels in resistant and susceptible plant varieties challenged with pathogens [80] and indicating the importance of polyamine metabolism in plant biotic stress responses, the precise mechanisms by which polyamines confer resistance to fungal pathogens remain unclear. One of the most widely accepted hypotheses is based on the ability of polyamines to bind to cell wall components, resulting in a strengthened physical barrier that prevents or limits fungal infection. A growing body of evidence also suggests that through oxidation and H_2_O_2_ generation, polyamines may act as mediators of plant defense activation [81]. In the present study, the putrescine hydroxycinnamyltransferase gene was shown to reach its highest levels of expression at 6 and 12 h after *Fusarium* inoculation in all genotypes tested, indicating that this gene is actively involved in defense response processes to infection by this pathogen (Table 4).

Peroxidases, encoded by the peroxidase 72 gene, are a superfamily of enzymes that catalyze the oxidation of various substrates using hydrogen peroxide as an electron acceptor. In plants, peroxidases are involved in many physiological processes, including cell wall lignification, suberization, and generation of reactive oxygen species (ROS), which, while potentially harmful, also serve as key signaling molecules that activate further defense responses [82].

Peroxidases contribute to pathogen resistance and protection against herbivores by strengthening cell walls, making them less susceptible to injury [83]. In addition, they can directly participate in defense against pathogens by generating toxic ROS or by cross-linking glycoproteins and other cell wall components to form a barrier against pathogens [83]. The peroxidase 72 gene encodes a specific isoform of peroxidase that has been shown to be induced after pathogen infection or injury [84]. This suggests that the peroxidase encoded by this gene plays a key role in the early stages of the plant defense response. Studies have shown that overexpression of the peroxidase 72 gene in transgenic plants leads to increased resistance to various pathogens, while silencing the gene results in increased susceptibility [78].

Etatricopeptide repeat (PPR) proteins, such as that encoded by At5g57250, are a large family of proteins characterized by tandem repeats of a 35-amino acid motif. PPR proteins are mainly located in mitochondria and chloroplasts, where they play an important role in RNA editing and splicing. In recent years, PPR proteins have also been implicated in plant defense signaling and resistance to various pathogens [85]. The At5g57250 protein is involved in maintaining chloroplast integrity under stress conditions. Chloroplasts, the site of photosynthesis, are also central centers of plant defense signaling. When plants encounter a pathogen or other stress, chloroplasts can release stress signals that activate further defense responses [85]. By ensuring proper chloroplast function, At5g57250 contributes to enhancing defense signals. While the peroxidase 72 gene and the At5g57250 protein have different functions, they likely work synergistically to enhance plant immunity. By modifying cell walls and generating ROS, peroxidases form the first line of defense against pathogens. Understanding the exact mechanisms by which these proteins contribute to plant immunity could pave the way for the development of crops with enhanced resistance to a wide range of stresses.

Lanubile et al. [68] showed that in resistant maize seedlings, before infection, ascorbate peroxidase expression was higher than in susceptible seedlings, and the enzyme was activated after pathogen infection. These results confirmed previous findings [86] regarding the primary defense response provided by maize genotypes resistant to F. *verticillioides* infection, both in kernels and seedlings. Lambarey et al. [87] investigated an African maize line, *Zea mays* CML144, infected with F. verticillioides. Genes associated with antioxidant activity were found within the list of genes associated with these GO terms. Within the matching up-regulated significant GO terms, the GRMZM2G427815 gene is associated with peroxidase and oxidoreductase activity. Peroxidase is one of the enzymes involved in scavenging reactive oxygen species (ROS) when the host is under pathogen attack or other stresses. This gene was also found to be expressed in both the resistant and susceptible genotypes. In the non-negative study, we observed that the expression levels of the peroxidase 72 gene and pentatricopeptide repeat-containing protein At5g57250 increase in each genotype after *Fusarium* inoculation. Interestingly, the highest expression level of both genes was observed in one of the subtypes 72 h after infection (Table 4). These results and literature references indicate active participation of peroxidase and pentatricopeptide repeat-containing protein At5g57250 in the resistant response to infection with a pathogen such as *Fusarium*. In particular, the peroxidase 72 gene is a promising candidate for a gene that can be linked to resistance to fusarium cucurbit. Current research, as well as previous reports in the literature, indicate that it can determine the resistance of plants to the pathogen.

Analysis of publicly available transcriptomic data revealed that the GDSL esterase/lipase gene At4g01130 precursor (carrying marker 553) shows the highest expression in Pericarp/Aleurone 27 DAP, the putrescine gene (carrying marker 15,097) has the highest expression in Primary root (5 days roots), while the peroxidase 72 gene (carrying marker 58771) shows the highest expression level in secondary roots. Only the putrescine gene does not undergo ubiquitous expression in both assimilating organs and various tissues. The strongest accumulation of mRNA occurs in roots for all genes studied (Figure 9). A similar observation was made by Tomkowiak et al. [65] in connection with the expression of genes related to maize yield.

In addition to checking the expression level of selected candidate genes in different maize genotypes and analyzing transcriptomic data, using Pearson’s coefficient method, the linear correlation of gene expression between individual genes was estimated (Figure 2), including basal metabolism genes. Candidate genes whose expression changes over time after inoculation occurred in correlated pairs: the putrescine gene and the peroxidase precursor gene, and each gene correlated with the basal metabolism gene. Similar correlations were observed in studies by Bocianowski [17] and Tomkowiak et al. [65].

Our study, consistent with previous reports, clearly demonstrates the role of these repulsion response genes in *Fusarium* infection. The molecular markers identified in their sequence can be used in breeding programs to select *Fusarium*-resistant maize genotypes.

## 5. Conclusions

The experiment showed a significant effect of both variety and time on the expression of the tested genes in maize. Expression analysis of candidate genes (*GDSL esterase/lipase*, *putrescine hydroxycinnamotransferase*, *peroxidase precursor 72*, and *uncharacterized protein*) showed that they had elevated expression levels after inoculation, indicating their involvement in the resistance response to infection by *Fusarium* spp.

The *peroxidase 72 precursor* was characterized by the highest level of expression after inoculation in all genotypes tested, which may predispose it to a gene potentially carrying resistance to *Fusarium* spp.

Genotypes from the same breeding company showed similar expression patterns, suggesting that they may be similar in origin.

## Figures and Tables

**Table 1:** Sequences of designed primers used to identify newly selected markers significantly associated with plant fusarium resistance genes.

Marker	Primer Sequences	Annealing Temperature (°C)	Product Size (bp)	Candidate Genes	Reference
553	F: TTGTCGACGTACACGACCG	60.0	116	*GDSL esterase/lipase gene* (LOC100273960)	[26]
	R: TTCGGGTGCGTGAAAAGCTA				
15,097	F: GGCTCACCTTCCCGTTCTAC	59.0	107	*Hydroxycinnamyltransferase gene* (LOC103649226)	[26]
	R: GTACGAAGGCACCAGGAACA				
58,771	F: TGCTAGCACAAGTGCATTTCAA	58.0	103	*Peroxidase 72 gene* (LOC100282124)	[26]
	R: TGAAGGTGTTGCAAGCGAAT				
27,775	F: ACCAGAAGAACATTCTGCAG	57.5	167	Gene encoding for an uncharacterized protein (LOC100501166)	[25]
	R: GAACGAGCTCACTCAGAAGC				

**Table 2:** Sequences and annealing temperatures (Tm) of primers used in PCR.

Gene Abbreviation	Gene Name	The Accession Number	Gene Symbol	Primer Sequence		Product Size on mRNA(bp)	Length Including Intron	Amplification Efficiency (%E)	R^2^	Tm (°C)
				Forward	Reverse					
F1	*GDSL esterase/lipase*	NM_001148347.1	LOC100273960	AAAGCGTCAAGCGAAGCCTA	GAACACCATGAAGCTGCGAG	98	224	123.9	1	60
F2	*Putrescine hydroxy cinnamyl transferase*	XM_008673542.3	LOC103649226	GTGGAGGTGGTGCAGGTG	CGAGGAAGTCGCTGGTGG	109	-	120.8	0.998	60
F3	Uncharacterized gene	NM_001195961.1	LOC100501166	GTGCGCTTGTCGTACTTCTTG	ACTACATAGGTAGGAGGAGCTCTA	105	207	93.8	1	60
F4	*Peroxidase 72* gene	NM_001155037.2	LOC100282124	ATTGTGCAGTCCATTGTGGC	GTTGTCCAACAGCACCGAAG	123	755	103.8	0.999	60
CYT	*Cyclophilin*	M55021/	LOC MZECYP	CTACCTCACGGCATCTGCTATGT	AACACGAATCAAGCAGAG	139	-	85.7	1	60
		Zm00001eb312970 (umc2057)								
β-TUB	β-tubulin	NP_001112117	LOC1920235	CTGAGTGGTGGTCTTAGT	GTCACACACACTCGACTTCACG	100	-	111.5	1	60

**Table 3:** Mean squares (m.s.) and *F*-statistics from repeated-measures univariate analysis of variance results for expression of six genes.

Source of Variation	d.f.	F1		F2		F3		F4		CYT	TUB		
		m.s.	*F*	m.s.	*F*	m.s.	*F*	m.s.	*F*	m.s.	*F*	m.s.	*F*
Line	4	0.0000011	18.18 ***	0.000053	16.85 ***	0.000048	9.03 **	0.000004	3.44	12.566	8.62 **	12.911	9.32 **
Residual	10	0.0000001	0.36	0.000003	0.97	0.000005	1.44	0.000001	0.85	1.457	0.5	1.385	0.79
Term	4	0.0000001	0.77	0.000039	12.03 ***	0.000077	20.73 ***	0.000003	1.83	18.904	6.43 **	20.51	11.69 ***
Term × Line	16	0.0000002	0.99	0.000007	2.16	0.000021	5.6 ***	0.000003	1.8	5.156	1.75	2.037	1.16
Residual	40	0.0000002		0.000003		0.000004		0.000001		2.94		1.755	

** *p* < 0.01; *** *p* < 0.001; d.f.—the number of degrees of freedom.

**Table 4:** Changes in expression levels after vaccination (relative to expression before vaccination—0 h) for four genes. Statistical test results are also presented—Kolmogorov–Smirnov test, Levene’s test, and Student’s *t*-test.

Line	Term	F1				F2				F3				F4			
		T/0 h	K-S Test	Levene’s	*t*-Student	T/0 h	K-S Test	Levene’s	*t*-Student	T/0 h	K-S Test	Levene’s	*t*-Student	T/0 h	K-S Test	Levene’s	*t*-Student
FR	00 h	1	0.916			1.000	0.770			1.000	0.928			1.000	0.696		
FR	06 h	2.916		0.026	0.963	10.608		0.024	0.249	10.667		0.053	0.252	17.565		0.039	0.756
FR	12 h	0.930		0.292	0.914	3.608		0.289	0.872	5.246		0.034	0.986	3.522		0.227	0.774
FR	24 h	1.168		0.236	0.226	1.351		0.287	0.001 ***	1.070		0.082	0.020 *	3.348		0.110	0.063
FR	72 h	2.608		0.192	0.303	2.284		0.105	0.561	5.807		0.059	0.199	0.304		0.037	0.932
KF12	00 h	1.000	0.987			1.000	0.898			1.000	0.892			1.000	0.702		
KF12	06 h	0.252		0.167	0.454	2.188		0.103	0.176	0.734		0.038	0.187	12.000		0.093	0.964
KF12	12 h	0.682		0.252	0.215	1.625		0.714	0.513	2.625		0.232	0.807	0.000		0.016 *	1.000
KF12	24 h	0.467		0.393	0.091	0.688		0.047	0.020 *	0.719		0.029	0.814	1.000		0.148	0.460
KF12	72 h	0.813		0.587	0.650	0.938		0.534	0.887	0.594		0.058	0.730	23.000		0.018	0.144
KF15	00 h	1.000	0.968			1.000	0.853			1.000	0.860			1.000	0.891		
KF15	06 h	1.583		0.046	0.673	4.961		0.065	0.094	6.142		0.095	0.450	17.000		0.057	0.650
KF15	12 h	0.777		0.442	0.589	2.169		0.036	0.534	0.206		0.111	0.563	4.000		0.078	0.472
KF15	24 h	0.709		0.066	0.290	0.590		0.090	<0.001 ***	0.394		0.397	<0.001 ***	6.000		0.022	0.053
KF15	72 h	1.524		0.040	0.344	0.680		0.035	0.625	2.277		0.022	0.234	0.000		0.158	0.891
SF11	00 h	1.000	0.949			1.000	0.954			1.000	0.939			1.000	0.933		
SF11	06 h	1.211		0.082	0.590	2.400		0.187	0.389	5.733		0.030	0.657	0.825		0.974	0.992
SF11	12 h	1.304		0.249	0.200	1.667		0.181	0.907	4.000		0.029	0.907	0.988		0.032	0.515
SF11	24 h	0.249		0.350	0.709	1.133		0.267	0.088	1.333		0.332	0.162	0.450		0.158	0.810
SF11	72 h	0.310		0.153	0.236	0.600		0.819	0.581	5.200		0.154	0.208	3.338		0.819	0.016 *
SF12	00 h	1.000	0.874			1.000	0.969			1.000	0.796			1.000	0.693		
SF12	06 h	0.230		0.067	0.182	1.916		0.128	0.942	4.074		0.104	0.369	6.667		0.022	0.995
SF12	12 h	0.409		0.501	0.196	1.050		0.105	0.758	0.360		0.225	0.745	0.667		0.636	0.997
SF12	24 h	0.430		0.264	0.091	0.780		0.179	0.213	0.771		0.249	0.001 ***	1.000		0.795	0.863
SF12	72 h	0.434		0.761	0.199	0.908		0.219	0.899	0.371		0.169	0.376	70.667		0.034	0.054

* *p* < 0.05; *** *p* < 0.001.

## Data Availability

The data presented in this study are available on request from the corresponding author.
